# The current state of hernia surgery and Brazilian hernia congress 2025

**DOI:** 10.3389/jaws.2026.16500

**Published:** 2026-06-19

**Authors:** P. H. F. Amaral, D. L. Lima, C. M. P. Claus, M. L. Furtado, P. H. F. Barros, L. M. Guimarães, P. Trauczinsky, S. Roll, L. T. Cavazzola, F. Malcher, H. M. G. Santos, G. Soares

**Affiliations:** 1 Department of Surgery, Santa Casa de São Paulo, São Paulo, Brazil; 2 Department of Surgery, Hospital Alemão Oswaldo Cruz, São Paulo, Brazil; 3 Department of Surgery, Montefiore Medical Center, New York, NY, United States; 4 Department of Surgery, Hospital Nossa Senhora das Graças, Curitiba, Brazil; 5 Department of Surgery, CIMi Institute, São Paulo, Brazil; 6 Department of Surgery, Hernia Clinic, São Paulo, Brazil; 7 Department of Surgery, Hospital da Fundação Oswaldo Aranha, Volta Redonda, Brazil; 8 Department of Surgery, Scolla – Centro de Treinamento em Cirurgia, Porto Alegre, Brazil; 9 Department of Surgery, Hospital Israelita Albert Einstein, São Paulo, Brazil; 10 Department of Surgery, Universidade Federal do Rio Grande do Sul, Porto Alegre, Brazil; 11 Department of Surgery, Lenox Hill Hospital, New York, NY, United States; 12 Hospital Universitário Ciências Médicas de Minas Gerais, Belo Horizonte, Brazil

**Keywords:** Brazil, hernia specialists, inguinal hernia, outcomes, ventral hernia

## BHS ERA 1 hernia surgery in Brazil and BHS summarized history

Over the years, abdominal wall surgery has traditionally been encompassed within the scope of general surgery. In recent decades, however, there has been growing recognition that abdominal wall defects present unique characteristics that warrant dedicated study and specialization. With this objective, the European and American Hernia Societies were established in 1979 and 1997, respectively, serving as pioneers for the creation of several other hernia-focused societies worldwide. In alignment with this global movement, the Brazilian Hernia Society (BHS) was founded in 2009.

In its early years, the BHS began with a limited number of members and encountered initial resistance, often questioned locally as to the necessity of a society dedicated exclusively to the study of abdominal wall hernias in Brazil. Its formative period was characterized by efforts to encourage surgeons to shift their perspective on hernia disease toward a more comprehensive approach, one that was more technically refined, dynamic, and inclusive of social and patient-centered considerations. Gradually, new members joined the society, following its development and motivated by the increasing prominence of international hernia societies. Concurrently, the rise of minimally invasive surgery (MIS) further accelerated engagement, attracting surgeons interested in mastering novel techniques initially demonstrated at international congresses and transforming established surgical practices.

Over its 15-year history, the BHS has played a central role in providing technical and scientific support to its members. Several clinical guidelines for inguinal and ventral hernia repair have been published, with the aim of guiding and adapting best practices in hernia surgery to the realities and resources of Brazilian healthcare. Currently, the BHS comprises approximately 524 active members, representing a 52% increase in membership over recent years.

## BHS ERA 2 – present: development and improving outcomes

### The evolution of abdominal wall surgery in Brazil and its current landscape

The geographical extent of Brazil and, most importantly, the presence of socioeconomic regions with markedly disparate realities poses significant challenges to the uniform delivery of surgical care across the country. In general, open surgical approaches continue to play a prominent role in the Brazilian healthcare setting and account for the majority of abdominal wall procedures performed nationwide. Open techniques have been systematically studied and documented since the 1960s, exemplified by the fascial transposition technique described by Professor Alcino Lázaro da Silva [1]. This contribution received well-deserved recognition and reflects Brazil’s longstanding commitment to the study and advancement of abdominal wall surgery.

Minimally invasive surgery (MIS) was introduced in Brazil in 1990 by Szego and Roll, with hernia surgery entering this field shortly thereafter in 1991 [2]. Although open surgery remains predominant, recent years have witnessed a progressive increase in the adoption of MIS techniques, particularly in major urban centers. Laparoscopic approaches have evolved substantially in Brazil, leading to internationally recognized contributions. Notable examples include the standardization of the transabdominal preperitoneal (TAPP) hernioplasty by Furtado and colleagues [3] and the development of an endoscopic technique for rectus diastasis repair by Claus, Malcher, and Cavazzola [4].

The first robotic abdominal wall procedure in Brazil was performed in 2008 by Abdalla and colleagues [5], at a time when only three robotic platforms were available nationwide and were primarily dedicated to urologic surgery. Over the past 5 years, the number of robotic systems has increased dramatically, with approximately 160 platforms currently in operation across the country. Robotic abdominal wall surgery has emerged as one of the fastest-growing fields and now occupies a prominent position in clinical practice. Techniques such as robotic transversus abdominis release (TAR), enhanced-view totally extraperitoneal (eTEP), and preperitoneal extended totally extraperitoneal (peTEP) repairs are now routinely performed by specialized teams. More recently, Brazil achieved a major milestone in telesurgery, with Cavazzola, Loureiro, and colleagues performing remote surgery over the longest reported distance between the surgeon console and the patient, measuring 12,034 km [6].

#### Brazilian data on hernia

Currently, approximately 400,000 abdominal wall surgeries are performed annually within the Brazilian public health system, representing a 13% increase in recent years [7]. Hernias account for a substantial proportion of patients awaiting surgical treatment in the public sector, largely due to the coexistence of high disease prevalence and prolonged waiting lists for access to care. Hernia disease represents a significant cause of work limitation and social security expenditures related to work absence, highlighting not only a healthcare burden but also an important social and humanitarian issue. The BHS plays a central role in patient and institutional awareness, contributing to informed decision-making by policymakers and fostering public investment in hernia care. In recent years, public funding dedicated to hernia treatment has increased by approximately 97 million US dollars.

#### Social activities

The BHS humanitarian surgical campaigns have been conducted for 11 years with the objective of promoting health through integrated clinical care and scientific engagement. Over this period, more than 1,000 patients have undergone surgical treatment, receiving comprehensive care from preoperative assessment through postoperative rehabilitation. These initiatives are carried out across different regions of the country, predominantly in remote or underserved areas, and aim to integrate local surgical teams while providing hands-on training and education. These campaigns typically reduce local hernia surgery waiting lists by 30%–50%.

#### Abdominal core health quality collaborative (ACHQC)

In an effort to better understand national outcomes in abdominal wall reconstruction surgery, the BHS initiated a partnership with the Abdominal Core Health Quality Collaborative (ACHQC) in 2022. Following necessary legal and regulatory adaptations to enable implementation of the ACHQC model in Brazil—supported by the ACHQC Board throughout the process—Brazil became the first country outside the United States to formally join the collaborative. Participation in the ACHQC enables benchmarking against international standards and fosters continuous quality improvement and maturation of surgical practice nationwide through clinical research.

#### Brazilian hernia congress 2025

The 2025 Brazilian Hernia Congress marked a milestone in BHS scientific events, representing the largest abdominal wall surgery meeting ever held in Brazil. The congress registered 543 participants, reflecting an approximate 30% increase compared with previous editions. This growth underscores the rising interest in abdominal wall surgery and the expanding credibility and positioning of the BHS. The scientific program featured 86 speakers from 13 countries and included 97 presented studies across inguinal, ventral, and hiatal hernia sessions, with dedicated tracks on preoperative optimization, surgical techniques, and management of postoperative complications.

## BHS ERA 3 – future perspectives

At the national level, two primary strategic objectives guide the BHS in the near future. The first is to capitalize on the significant evolution of hernia and abdominal wall surgery and to formally establish it as an independent and recognized surgical subspecialty in Brazil, rather than within general surgery. When this goal is achieved, the BHS will be fully prepared to support residency training programs in the structured education and certification of hernia specialists.

The second objective relates to ongoing efforts to improve reimbursement for abdominal wall surgeons within both public and private healthcare systems. Currently, there is limited stratification of procedural complexity, which may result in reimbursement for primary, low-complexity hernia repairs being unjustly similar to that of complex abdominal wall reconstructions. These procedures require distinct levels of technical expertise, differ substantially in operative complexity and postoperative course, and are associated with significantly higher operational costs.

From an international and academic perspective, the BHS aims to continue providing its members with technical guidance, education, and scientific support to ensure the delivery of high-quality abdominal wall surgery. The long-term objective is to align Brazilian practice standards with those of the most established and influential hernia societies worldwide.

The Brazilian Hernia Society is a relatively young, socially engaged organization that has demonstrated consistent growth over the last decade. This trajectory was exemplified by the 2025 Brazilian Hernia Congress, the largest and most impactful abdominal wall surgery meeting ever held in Brazil. The BHS envisions a future in which hernia and abdominal wall surgery is formally recognized as a distinct medical specialty, reflecting its complexity, scientific maturity, and clinical relevance.

## Data Availability

The original contributions presented in the study are included in the article/supplementary material, further inquiries can be directed to the corresponding author.
